# Exploration of Volatileomics and Optical Properties of *Fusarium graminearum*-Contaminated Maize: An Application Basis for Low-Cost and Non-Destructive Detection

**DOI:** 10.3390/foods13193087

**Published:** 2024-09-27

**Authors:** Maozhen Qu, Changqing An, Fang Cheng, Jun Zhang

**Affiliations:** 1 College of Biosystems Engineering and Food Science, Zhejiang University, Hangzhou 310027, China; 12313001@zju.edu.cn (M.Q.); 12213004@zju.edu.cn (C.A.); 2 College of Mechanical and Electrical Engineering, Jiaxing Nanhu University, Jiaxing 314001, China

**Keywords:** asymmetric polarization, *F. graminearum*-contaminated maize, optical imaging platforms, optical properties, spectroscopy system, volatileomics

## Abstract

*Fusarium graminearum* (*F. graminearum*) in maize poses a threat to grain security. Current non-destructive detection methods face limited practical applications in grain quality detection. This study aims to understand the optical properties and volatileomics of *F. graminearum*-contaminated maize. Specifically, the transmission and reflection spectra (wavelength range of 200–1100 nm) were used to explore the optical properties of *F. graminearum*-contaminated maize. Volatile organic compounds (VOCs) of *F. graminearum*-contaminated maize were determined by headspace solid phase micro-extraction with gas chromatography-tandem mass spectrometry. The VOCs of normal maize were mainly alcohols and ketones, while the VOCs of severely contaminated maize became organic acids and alcohols. The ultraviolet excitation spectrum of maize showed a peak redshift as fungi grew, and the intensity decreased in the 400–600 nm band. Peak redshift and intensity changes were observed in the visible/near-infrared reflectance and transmission spectra of *F. graminearum*-contaminated maize. Remarkably, optical imaging platforms based on optical properties were developed to ensure high-throughput detection for single-kernel maize. The developed imaging platform could achieve more than 80% classification accuracy, whereas asymmetric polarization imaging achieved more than 93% prediction accuracy. Overall, these results can provide theoretical support for the cost-effective preparation of low-cost gas sensors and high-prediction sorting equipment for maize quality detection.

## 1. Introduction

Due to the rich nutrients, maize is susceptible to *Fusarium* spp. (*F.* spp.) infection during growth [1,2], which leads to ear rot. Mycotoxins, as metabolites of fungi, accumulate in maize kernels, posing a potential threat to the health of humans and poultry [3]. Deoxynivalenol (DON) and zearalenone (ZEN) in mycotoxins are key indicators to measure the quality of maize, which are both rated as class III carcinogens by the International Agency for Research on Cancer [4]. It is worth noting that both DON and ZEN can be metabolized by *F. graminearum* species complex (FGSC) [5,6]. Under low temperatures, *F. graminearum* is more likely to become the dominant fungus of maize ear rot [7,8].

The evaluation methods of fungal contamination in food mainly included polymerase chain reaction (PCR) [9], colony counting [10], and fungal spore counting [11]. The detection of fungi was labor-intensive, and the results were subjective, which were not conducive to the assessment of on-site fungal contamination. [12,13,14]. Moreover, fungal contamination was positively correlated with its metabolite mycotoxins, which could be detected quickly and conveniently [15]. Thus, people often used mycotoxin concentrations as the key indicator to measure food safety. In practical applications, the detection methods of mycotoxins included high-performance liquid chromatography, enzyme-linked immunosorbent assays (ELISA) [16], colloidal gold immunochromatographic assays, and fluorescence-labeled immunochromatography [17]. Due to the cumbersome and high cost of the detection process, the number of sampling tests was limited, which could lead to food waste or mycotoxin recontamination.

Researchers gradually favored non-destructive detection methods due to the advantages of rapid and low-cost detection of fungi and fungal metabolite mycotoxins, including optical and volatile organic compounds (VOCs) detection methods [18,19]. Optical detection technology was widely used in grain quality detection, such as hyperspectral imaging [20], Raman spectroscopy [21], and ultraviolet/visible/near-infrared (UV/VIS/NIR) spectroscopy [22,23]. Hyperspectral imaging could record the spatial and spectral information of grains [24], which helped to identify mycotoxin-contaminated grains and accurately distinguish different fungal colonies quickly. The acquisition of rich features made it easy to combine hyperspectral data with deep learning algorithms for accurate classification of fungal-contaminated grains [25]. Due to the high cost of equipment, hyperspectral technology was difficult to apply for the detection of large quantities of fungal-contaminated maize. Raman spectroscopy was sensitive to fungal infection and had a good response to fungal hyphae [26]. However, Raman spectroscopy encountered some challenges in the detection of single grains. UV/VIS/NIR spectroscopy could detect the contamination of various fungi and mycotoxins in maize [27,28], and the presence of fungi had a significant effect on the spectral curve. However, due to the limited performance of low-cost hardware, many optical technologies were difficult to apply in practice. With the development of photosensitive chips, low-cost industrial cameras have good response signals for VIS/NIR bands [29]. At the same time, Aflatoxin B1 and ZEN could produce a fluorescence effect or peak shift in the VIS band under UV excitation [30,31]. Thus, a thorough study of the optical properties of fungal-contaminated maize that low-cost photosensitive chips can capture may help to improve the prediction accuracy of current grain color sorters. Meanwhile, the rational use of polarization technology can enhance the visual difference between fungal-contaminated and normal grains by means of light transmission and polarization mirror characteristics [32,33]. Accordingly, the optical properties of *F. graminearum*-contaminated maize in the UV/VIS/NIR band are important theoretical support for the development of low-cost, high-throughput, and high-precision sorting equipment.

The growth of fungi is accompanied by the production of VOCs. Detection methods for VOCs included gas chromatography-tandem mass spectrometry (GC-MS) [34], gas chromatography-ion mobility spectrometry (GC-IMS) [35], electronic nose [36], and artificial olfactory sensors (AOS) [37]. GC-MS and GC-IMS could accurately identify and quantify VOCs [38], which were commonly used as standard methods for detecting VOCs. The potential VOCs of maize ear rot infected by *F. graminearum* were mainly heptan-2-ol, 1-octen-3-ol, and octan-3-one through GC-MS [39]. In addition, the electronic nose could detect VOCs of fungal-contaminated maize to achieve maize quality detection, such as *Aspergillus*-contaminated maize, *Penicillium*-contaminated maize, and *Fusarium*-contaminated maize [40,41]. Moreover, AOS was a portable and cost-effective technique consisting of chemical dyes and solid support [42], and chemical dyes could produce different reactions with different color changes [43,44,45]. AOS has demonstrated excellent ability to evaluate *Aspergillus*-contaminated maize [46] and had good potential to identify fungal-contaminated plants [47], showing excellent application potential at lower preparation costs. The harvested maize did not contain maize cob and maize ear, which may lead to the difference between VOCs of fungal-contaminated maize kernels and VOCs of maize ear rot. Furthermore, the analysis of the VOCs difference between fungal-contaminated maize and normal maize could provide a good basis for applying AOS. Therefore, understanding the volatileomics of *F. graminearum*-contaminated maize is helpful in identifying the main VOCs to develop AOS that is more suitable for maize quality detection.

Fungi and mycotoxins have some effects on maize’s optical properties, and fungi are the key factors affecting the volatileomics of maize. Since post-harvest maize is often contaminated by *F. graminearum*, exploring the optical properties and volatileomics of *F. graminearum*-contaminated maize is crucial for developing non-destructive detection equipment that ensures grain safety. Herein, the reflection optical properties and transmission optical properties of maize were explored through the self-designed reflection spectroscopy systems, and the characteristic VOCs of *F. graminearum*-contaminated maize were identified by GC-MS. Furthermore, the reflection and transmission imaging platforms were developed to verify the application feasibility of the optical properties of *F. graminearum*-contaminated maize. Finally, asymmetric polarization imaging was proposed as a method that combined transmission optical properties and reflection optical properties.

## 2. Materials and Methods

### 2.1. Materials

*F. graminearum CICC 2697* was obtained from Ningbo Testobio Co., Ltd. (Ningbo, China), which was isolated from the stem-rotting plant and preserved in the China Center of Industrial Culture Collection (https://cicc.china-cicc.org/). Glucose (analytical pure), AGAR powder (biological reagent), and sodium hypochlorite (analytical pure, NaClO) were purchased from Sinopharm Chemical Reagent Co., Ltd. (Shanghai, China), Nanjing Quanlong Biotechnology Co., Ltd. (Nanjing, China), and Guangdong Wenglong Chemical Reagent Co., Ltd. (Shaoguan, China). Maize was obtained from the natural golden fruit seed industry (Shouguang, China). The maize variety is *Zhengdan 958*, which is widely cultivated in China.

### 2.2. Preparation of F. graminearum Spores

*F. graminearum* was transferred to potato dextrose agar (PDA) medium for strain activation, and the untreated PDA medium was used as a control group to ensure that microorganisms did not contaminate the medium. The medium was sealed and placed in an incubator at 25 °C. After seven days of constant temperature culture, fungal hyphae spread to the edge of the medium, and no colonies appeared in the control medium. Subsequently, the medium was punched to obtain a fungus dish containing hyphae, and the fungus dish was transferred to the mung bean soup medium with tweezers. The mung bean soup medium was placed in an alternating light and dark environment and was shaken at 25 °C for five days to obtain fungal spores. After the culture was completed, the mung bean soup medium containing fungal spores was filtered through sterile gauze to obtain the filtrate, and the filtrate was centrifuged for 10 min at 6000 rpm. After centrifugation, the sediment was mixed with sterile water to obtain a fungal spore concentration of 1 × 10^6^ CFU/mL.

### 2.3. Preparation of Maize Samples

Firstly, all maize samples were soaked in 10% sodium hypochlorite for 1 min to kill bacteria on the surface of the maize. Then, sterile water was used to rinse the maize five times to remove sodium hypochlorite and fungal spores from the maize surface. After natural drying, 80 g of maize was evenly mixed with 1 mL of fungal spore solution and transferred to a sterile culture flask. It is worth noting that the number of fungal spores in maize was more than 1 × 10^4^ CFU/g. The moisture content of maize was adjusted from 13% to 22% to provide sufficient moisture for fungal spore growth. The above experimental operations were carried out in the XinNiu ultra-clean workbench (XinNiu, Guangzhou, China). The flask was sealed with PM996 film (Parafilm, Washington, DC, USA) and stored in an artificial climate chamber (25 °C) to simulate the growth process of *F. graminearum* in maize. Maize inoculated with fungal spores was stored in an incubator that alternated between light and dark, and the storage time was divided into five sample points (i.e., 0, 10, 18, 24, and 30 days) corresponding to samples 1 to 5. Sample 1, not inoculated with *F. graminearum*, was stored in a refrigerator at 4 °C as a control group. Sample 1 was further divided into sample 1 (untreated), sample 1 (sterilized with NaClO), and sample 1 (cultured for five days), where sample 1 (cultured for five days) had the same experimental procedure (sterilized, sealed, and stored at constant temperature) as samples 2 to 5. Three sample 1s were prepared to investigate the effects of NaClO oxidation in detail. After the culture was completed, the moisture content of each group of samples would be reduced to less than 15% and stored in a refrigerator at 4 °C.

### 2.4. Evaluation for F. graminearum-Contaminated Maize

#### 2.4.1. Flat Colony Counting Method

Fungal spores are constantly produced during fungal growth. Thus, the number of spores can be used as an important indicator of fungal contamination. According to the national standard [48], the detection of fungi in maize can be achieved by spore counting. A total of 10 g maize samples were mixed with 30 mL sterile water. After 1 min of violent oscillation, the spore suspension was filtered by sterile gauze. The number of fungal spores was determined by a blood cell counting plate.

#### 2.4.2. DON and ZEN Concentration Measurements

*F. graminearum* continuously produces metabolic toxins (DON and ZEN) during its growth. Therefore, DON and ZEN concentrations were measured by ELISA as two important reference indicators of *F. graminearum* contamination. The ELISA kits for ZEN and DON were purchased from Wuhan Huamei Wishercon Bioengineering Co., Ltd. (Wuhan, China), which passed the applicability verification of mycotoxins in the testing center certified by the National Food and Material Reserve Bureau. The specific operation process is as follows: (1) 50~100 g maize samples were crushed and passed through a 20-mesh screen (particle size < 1 mm), and the powder was mixed evenly. A total of 5 g ± 0.05 g maize was weighed and mixed with 25 mL ethanol (40%), followed by shaking for 2 min. (2) After centrifugation, a certain amount of supernatant was mixed with the diluent to obtain dispersion, in which DON and ZEN were diluted by 100 and 200 times, respectively. (3) After calibration, 5 μL dispersion was added to the corresponding micropores, and then 50 μL enzyme conjugate and 50 μL antibody were added, respectively. After sealing the micropores, the mixture was mixed and placed at 25 °C for 15 min. (4) The mixture in the micropores was poured out, and the micropores were washed several times. The micropores were added to a 100 μL substrate solution and colored at 25 °C for 5 min. Subsequently, 50 μL of the termination solution was dropped into the micropores. (5) The concentration of DON or ZEN could be calculated using a microplate reader (wavelength: 450/630 nm).

### 2.5. Headspace Solid Phase Micro-Extraction with GC-MS (HS-SPME-GC-MS)

#### 2.5.1. HS-SPME

A total of 3 g of maize was weighed and placed in a 20 mL headspace sample bottle, which was sealed with tin foil. After the cap was tightened, the headspace sample bottle was placed in a 45 °C water bath and stirred magnetically at 100 rpm for 10 min. A 50/30 μm divinylbenzene/carboxen/polydimethylsiloxane extraction needle was heated to 250 °C and maintained for 30 min to remove impurities, and then the injector was inserted into the sample bottle. The fiber extraction needle was exposed to VOCs above the maize and contacted with VOCs at 45 °C for 40 min. After the adsorption of VOCs was completed, the fiber extraction needle was taken back into the sampler.

#### 2.5.2. GC-MS

A 7890B gas chromatograph (Agilent, Santa Clara, CA, USA) with the 5977A mass selective detector (Agilent, Santa Clara, CA, USA) was used to analyze HS-SPME samples. The fiber extraction needle was quickly inserted into the inlet of the gas chromatograph and exposed to achieve pyroadsorption. The HP-5 ms chromatographic column (30 m × 250 μm × 0.25 μm, Agilent) was used to analyze VOCs. The GC inlet temperature was set at 250 °C, and the non-shunt sampling method was adopted. The heating program was set at 40 °C for 2 min, followed by a rise to 140 °C at 3.2 °C·min^−1^. Then, the temperature rose from 140 to 250 °C at 8 °C·min^−1^. The carrier gas was high-purity helium (99.999%), and the flow rate was maintained at 1.0 mL/min. The scanning mass range was 40–450 atomic mass units.

### 2.6. Optical Properties Analysis

#### 2.6.1. Reflectance Spectroscopy System (RSS)

**Figure 1:**
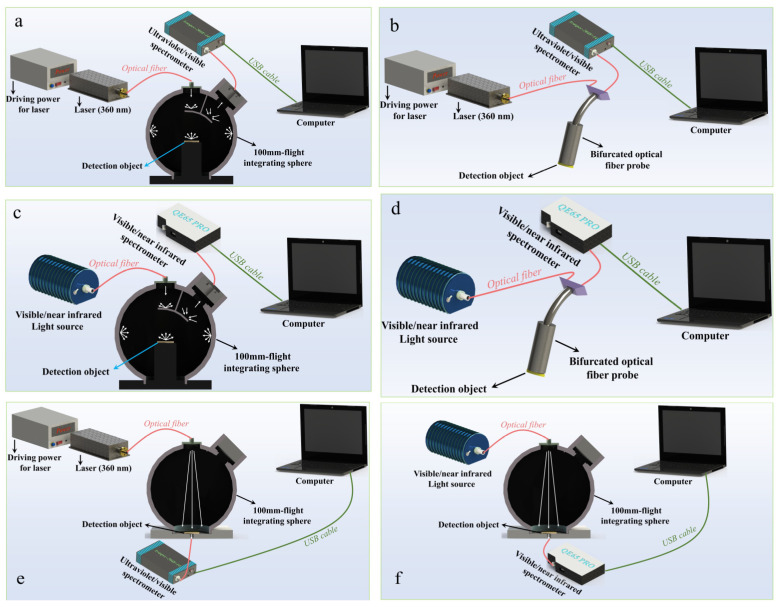
RSS-1 (**a**) and RSS-2 (**b**) containing a UV laser. RSS-1 (**c**) and RSS-2 (**d**) with VIS/NIR light source. TSS containing a UV laser (**e**) and a VIS/NIR light source (**f**).

Reflection optical properties of *F. graminearum*-contaminated maize were explored by RSS under different excitation light sources and reflection modes. One was the RSS-1 based on the integrating sphere, and the other was the RSS-2 based on the bifurcated optical probe. The incident band of the light source covered the UV and VIS/NIR bands. For RSS-1 with a UV light source (Figure 1a), the system was mainly composed of a UV laser (MW-GX-360, Hangzhou, China), a laser drive power supply, a spectrometer (Avantes, Holland), a 100 mm integrating sphere (Flight, Hangzhou, China), and a computer. As shown in Figure 1b, the integrating sphere in the RSS-2 with an UV light source was replaced by a bifurcated fiber optical probe (Ocean Optics, Orlando, FL, USA). For RSS-1 with VIS/NIR light source (Figure 1c), the system consists of a VIS/NIR light source (Ocean Optics, USA), a spectrometer (Ocean Optics, USA), a 100 mm integrating sphere, and a computer. Similarly, the integrating sphere conversion was also replaced with a bifurcated fiber optical probe in the RSS-2 of the VIS/NIR light source (Figure 1d).

The light of different bands was uniformly irradiated to the detection object through the surface coating of the integrating sphere, and the light would be reflected from the surface of the detection object into the optical fiber. The hole around the center of the bifurcated fiber optical probe would emit light in the corresponding band, and the center hole would receive the light reflected by the maize. The bifurcated fiber probe could generate smaller spots and higher energy density than the integrating sphere. Therefore, the two reflection methods were combined to better analyze the reflective optical properties of *F. graminearum*-contaminated maize.

#### 2.6.2. Transmission Spectroscopy System (TSS)

Both TSS and integrating sphere-based RSS-1 had the same components. As shown in Figure 1e,f, the difference was that the optical fiber connecting the spectrometer in TSS was located directly below the detection object. After the light of different bands passed through the detection object, the spectrometer would receive the transmitted light. The transmission optical properties of *F. graminearum*-contaminated maize were determined by analyzing the difference in light signals.

### 2.7. Design of Rapid Detection Platforms for F. graminearum-Contaminated Maize

#### 2.7.1. Optical Reflection and Transmission Imaging Platform Based on Optical Properties of *F. graminearum*-Contaminated Maize

**Figure 2:**
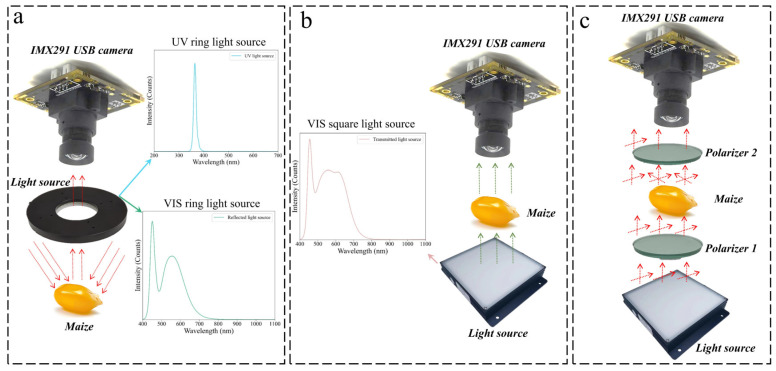
Composition of (**a**) reflection imaging platform, (**b**) transmission imaging platform, and (**c**). asymmetric polarization imaging platform.

The optical reflection platforms mainly consisted of a USB camera equipped with the IMX291 sensor (SONY, Tokyo, Japan), a UV or VIS ring light source, and a machine vision test bench. As shown in Figure 2a, the camera was located in the center of the ring light source to receive the optical signal reflected from the maize surface. The UV ring light source (band range of 350–370 nm) and the VIS ring light source (main band range of 450–650 nm) were used to obtain the optical signal of the maize surface and were used as the core light source for UV excitation reflection imaging and VIS reflection imaging, respectively. The optical transmission platform (Figure 2b) was mainly composed of a USB camera equipped with the SONY IMX291 sensor, a square light source of VIS light (main band range of 450–700 nm), an optical glass sheet, and a machine vision test bench. The camera was mounted directly above the square light source, and the optical glass sheet existed between the light source and the camera. The maize was placed on the upper surface of the optical glass sheet. The light from the square light source passed through the maize kernel and was received by the photosensitive chip in the camera.

Before data collection, the power of the three light sources was set to a constant value, and the optical parameters of the camera remained consistent until the end of the experiment. For each group of samples, 30 feature images were randomly collected from the endosperm surface and germ surface. A total of 300 feature images were collected from each optical platform. A total of 900 images were captured from three optically reflected platforms.

#### 2.7.2. Asymmetric Polarization Imaging Platform Based on Transmission and Reflection Optical Properties

The composition of the asymmetric polarization imaging platform was similar to that of the TSS. The difference was that the asymmetric polarization imaging platform added two linear polarizers. As shown in Figure 2c, a polarizer was mounted directly above the light source, and a polarizer was also mounted directly below the camera. When the polarizer 1 was fixed, light signals in different directions could be effectively screened by rotating the polarizer 2. The maize was placed between two polarizers, and the camera received the light signal transmitted from the maize that was enhanced by the signal of the polarizers. In order to combine the optical reflection and transmission properties, two polarizers were fixed in a non-completely parallel way. It was worth noting that the light signal in the center of the polarizers could not pass through when the maize was not placed, and the light signal intensity would increase with the increase of the center of the circle.

Therefore, asymmetric polarization was expected to enhance transmitted signals and acquire surface optical signals. The asymmetric polarization imaging platform and optical transmission platform had the same light source power, camera parameters, maize signal acquisition surface, and the number of feature images.

### 2.8. Feature Extraction Method

In the BGR color space, channel B was a blue color channel, channel G was a green color channel, and channel R was a red color channel. Channel B, channel G, and channel R were selected for color feature analysis. In view of the large number of features, the color moment (mean, variance, and skewness) was used to extract the color distribution of the kernel surface in maize, written as follows:

(1) $$Mean_{i}=\frac{1}{X}\frac{1}{Y}\sum_{x=1}^{X}\sum_{y=1}^{Y}channel_{i}(x,y)$$

(2) $$Variance_{i}={\left(\frac{1}{X}\frac{1}{Y}{\sum_{x=1}^{X}\sum_{y=1}^{Y}\left(channel_{i}(x,y)-mean_{i}\right)}^{2}\right)}^{\frac{1}{2}}$$

(3) $$Skewness_{i}={\left(\frac{1}{X}\frac{1}{Y}{\sum_{x=1}^{X}\sum_{y=1}^{Y}\left(channel_{i}(x,y)-mean_{i}\right)}^{3}\right)}^{\frac{1}{3}}$$

where *channel_i_*(*x*, *y*) is the color intensity in the *x*, *y* coordinate in the *i* channel; and *X*, *Y* are the maximum value of the abscissa and ordinate, respectively.

### 2.9. Statistical Analysis

Python programs and external packages (sci-kit-Learn 1.0.2, NumPy 1.21.5, etc.) were used to train models and predict contamination levels, and SPSSPRO (Version 1.0.11) was used for the significance test. Firstly, nine color feature vectors were used for vector normalization, and the normalized data were divided into a training set and a prediction set according to a ratio of 7:3. Then, principal component analysis (PCA) and support vector machine (SVM) were used for modeling to evaluate the feasibility of the imaging method.

Since PCA was an unsupervised classification model, both the training set and the prediction set were used for PCA modeling. The calculation process of PCA was as follows: (1) All data needed to be sample-centered by subtracting the mean value of each vector from each vector (Formula (4)). (2) The covariance matrix was calculated using the obtained centered vector (Formula (5)), and the eigenvalues and eigenvectors of the covariance matrix were solved. (3) The eigenvector corresponding to the maximum *n* eigenvalues was obtained, and the eigenvector matrix *W^T^* was constructed after the eigenvector was normalized. (4) The original sample was converted into a new sample vector by using Formula (6), and the output data set was obtained by combining all the new sample vectors.

(4) $$x(i)=x(i)-\frac{1}{N}\sum_{j=1}^{N}x(j)$$

(5) $$Cov(x,y)=\frac{\sum_{n=1}^{N}\left(x_{n}-\bar{x})(y_{n}-\bar{y}\right)}{N-1}$$

(6) $$z(i)=W^{T}x(i),\;W^{T}=(w_{1},w_{2},\ldots ,w_{N})$$

(7) $$D_{output}=(z_{1},z_{2},\ldots ,z_{N})$$

where *x*, *y* are the original data vector or the sample-centered vector; *Cov*(*x*, *y*) represents the calculation process of covariance values; *Z* and *W^T^* represent new sample vectors and coefficient matrices; and *D_output_* represents the data set after dimensionality reduction.

The calculation principle of SVM is to find a hyperplane to maximize the interval between the two levels so as to achieve a good classification effect, and SVM is a supervised classification model. The hyperplane is *wx* + *b* = 0, and the data points closest to the hyperplane need to be solved (Formula (8)). Therefore, it is very important to find a hyperplane with the largest interval, and the optimization problem needs to be solved in Formula. The linear function kernel (Formula (9)) was used to solve the hyperplane to establish an evaluation model for *F. graminearum*-contaminated maize.

(8) $$\min_{w,b}\frac{1}{2}{\left||w|\right|}^{2}$$

(9) $$k(x,x_{i})=xx_{i}$$

where *w* is the vector, and *b* is a constant.

## 3. Result and Discussion

### 3.1. Evaluation of Maize Quality with the Growth of F. graminearum

**Figure 3:**
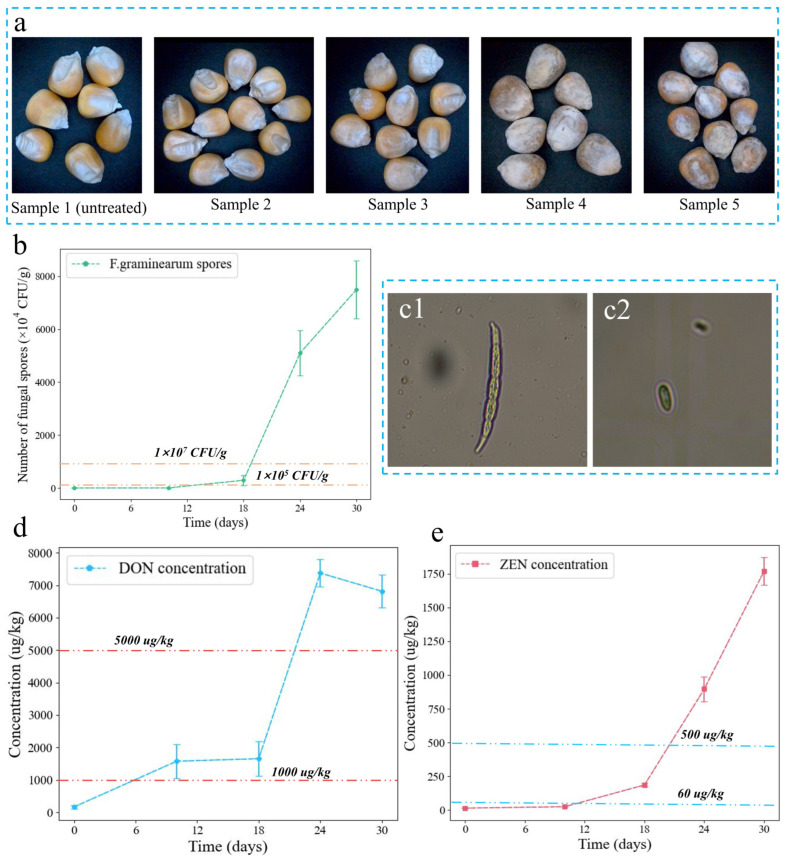
(**a**) Images of maize samples with different levels of *F. graminearum* contamination. (**b**) Change curves of fungal spores in maize with the growth of *F. graminearum*. *F. graminearum* spores observed in PDA medium (**c1**) and maize (**c2**) after magnified 400 times. Change curves of DON concentration (**d**) and ZEN concentration (**e**) in maize with the growth of *F. graminearum*.

Five maize samples were shown in Figure 3a. With the increase of culture time, the fungal hyphae on the surface of maize became more and more obvious, resulting in significant mildew characteristics. Five maize samples were measured for fungal spore count, DON, and ZEN concentrations to quantify the effect of *F. graminearum* on maize quality. From Figure 3b, the number of spores gradually increased from 1 × 10^4^ CFU/g to 7.5 × 10^7^ CFU/g after 30 days, indicating that the fungal hazard level gradually changed from safe to serious. It is worth noting that the spores of *F. graminearum* in the mung bean soup medium were mainly large sickle-shaped spores (Figure 3(c1)), while the spores produced by *F. graminearum* in maize were mainly smaller conidia (Figure 3(c2)). DON concentration increased from 169.17 μg/kg to 6813.3 μg/kg after 30 days, and ZEN concentration increased from 14.53 μg/kg to 1767.72 μg/kg after 30 days (Figure 3d,e), which has seriously exceeded the maximum residual residue of mycotoxins in the feed standards of most countries. From the above analysis, it can be seen that the number of spores and the concentration of mycotoxins showed a similar change law, which increased slowly at first and then increased rapidly. Through the analysis of the three indicators, sample 1 was classified as normal, samples 2 and 3 were classified as mildly contaminated, and samples 4 and 5 were classified as severely contaminated.

### 3.2. Analysis of Characteristic VOCs of Maize in Different Stages of F. graminearum Contamination

**Figure 4:**
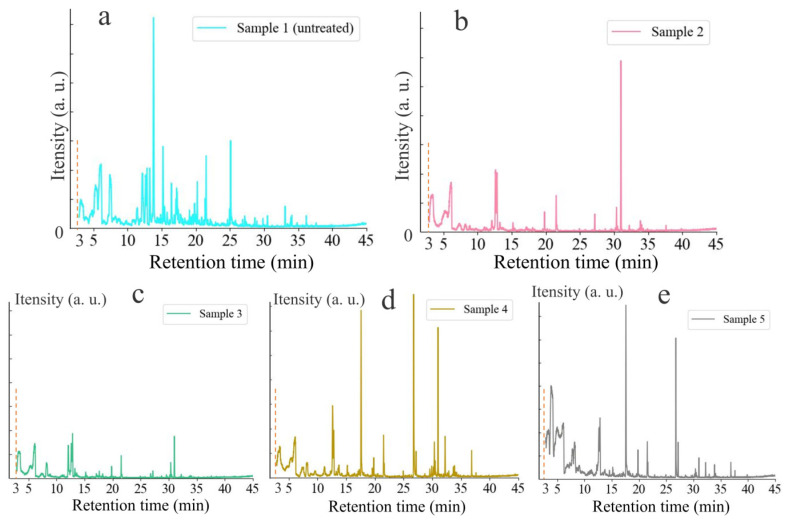
Detection of VOCs released from sample 1 (**a**), sample 2 (**b**), sample 3 (**c**), sample 4 (**d**), and sample 5 (**e**) by HS-SPME-GC-MS.

As depicted in Figure 4, the number of VOCs released from maize decreased first and then increased according to the number of peaks in the chromatogram with the growth of *F. graminearum*. Compared with the other samples (Figure 4a–e), sample 3 produced the least number of peaks in the chromatogram, and the peak area was relatively small, indicating that the VOCs of sample 3 were the least. With the growth of the hyphae, *F. graminearum* would consume the nutrients of maize and produce more and more characteristic VOCs of *F. graminearum*. However, the loss of nutrients and the death of maize would lead to a decrease in the number of maize-specific VOCs. In order to find out the VOCs released by maize at different stages of *F. graminearum* contamination, the VOCs collected by the mass spectrometer were retrieved by the NIST20.L library to determine the composition and relative content of VOCs. Since the VOCs from samples 2 to 4 were small in quantity and concentration, HS-SPME-GC-MS was used to detect VOCs released from sample 1 (normal maize) and sample 5 (severely contaminated maize). Due to the low concentration of VOCs produced by maize, some siloxanes from the chromatographic column may be detected. After excluding the VOCs consistent with the blank samples, 49 VOCs were detected in normal maize and 19 VOCs were detected in severely contaminated maize through the retrieval and analysis of the characteristic peaks of the full spectrum. There were 11 VOCs with the highest relative content and that met the matching factor (≥78%). Subsequently, n-alkanes containing C8–C14 were used to calculate the retention index. Compared with the RI in the NIST database, the allowable error range of the RI of VOCs in this study was ±5%. Thus, VOCs that meet the matching factor and RI were shown in Table 1 and Figure S1. The remaining organic compounds (low relative content, did not meet the matching factor, had a large RI error, or did not belong to the common organic compounds) were supplemented in the Supplementary Information (Table S1). For normal maize, the main VOC markers included alcohols, ketones, aldehydes, and aromatic organic compounds, among which alcohols mainly belonged to fatty alcohols. For severely contaminated maize, the main VOC markers included carboxylic acid organic compounds, alcohols, ethers, aromatic organic compounds, aldehydes, and ketones, among which carboxylic acid organic compounds were mainly acetic acid. With the increase of *F. graminearum* contamination, the concentration of fatty alcohols gradually decreased, and the concentration of carboxylic acid organic compounds gradually increased. The increase in acid substances may be related to a humid environment and poor air fluidity. In previous studies, the characteristic VOCs of maize contaminated by *Fusarium* and *Aspergillus* were dominated by alcohols, aldehydes, and ketones [39,49,50], and the characteristic VOCs of maize ear rot contaminated by *F. graminearum* were dominated by alcohols and sesquiterpenes. In this study, *F. graminearum* may accelerate the decomposition of carbohydrates in maize under limited oxygen concentration due to poor air mobility, resulting in an increase in organic acids. The poor air fluidity simulated that maize was in a low concentration of oxygen environment in wet weather. The changes in the concentrations of alcohols and organic acids indicated that it was feasible to determine the contamination level of *F. graminearum* in maize by VOCs. Remarkably, some alcohols can be perceived by the human olfactory system at room temperature, such as 1-pentanol, 3-methyl-, and acetic acid. Therefore, the volatiliomics of *F. graminearum*-contaminated maize can provide theoretical support for the development and practical application of gas sensors for maize quality detection.

### 3.3. Effects of Sterilization on Optical Properties of Maize

**Figure 5:**
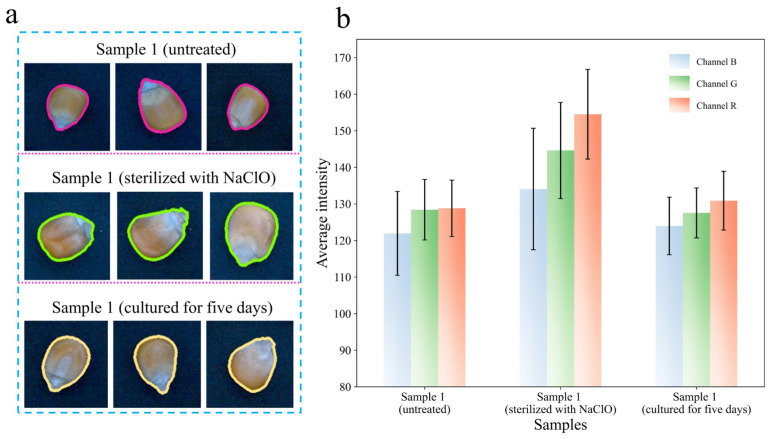
(**a**) Image of sample 1 (untreated), sample 1 (sterilized with NaClO), and sample 1 (cultured for five days). (**b**) Average color intensity of the three samples.

NaClO can almost eliminate all bacteria and does not affect seed vigor by virtue of its oxidability, but it may have a slight effect on the surface color of maize and affect the optical properties of the maize at the beginning of fungal growth [51,52]. In order to explore the effect of NaClO on maize, three sample 1s (a total of 150 maize kernels) were used for the difference analysis of optical properties. Through Figure 5a, three samples were extracted from the regions of interest of the image to calculate the average color intensity of the B, G, and R channels. The average color intensity of the three channels and its standard deviation were calculated and shown in Figure 5b, where it can be observed that the average color intensity of sample 1 (sterilized with NaClO) was higher than that of sample 1 (untreated) and sample 1 (cultured for 5 days).

Considering the complete independence between any two samples, the Mann–Whitney test was used to quantify the differences between the three samples based on the average color intensity of B, G, and R channels, as shown in Table 2. When sample 1 (untreated) and sample 1 (cultured for 5 days) were a group of independent samples, the *p*-value was less than 0.05 for channel B, channel G, and channel R, indicating that there was no significant difference between them. When constructing independent samples of sample 1 (sterilized with NaClO) and sample 1 (untreated) or sample 1 (cultured for 5 days), the *p*-values on channel R, channel G, and channel B were all greater than 0.05, which proved that there were significant differences between sample 1 (sterilized with NaClO) and the other two samples. Cohen’s d value can quantify differences by thresholds of 0.2 (small difference), 0.5 (medium difference), and 0.8 (large difference). Cohen’s d values of independent samples constructed on the B, G, and R channels for sample 1 (untreated) and sample 1 (cultured for 5 days) were 0.204, 0.112, and 0.261, respectively. Thus, there was a small difference in optical properties between sample 1 (untreated) and sample 1 (cultured for 5 days). In the independent samples containing sample 1 (sterilized with NaClO), the maximum and minimum values of Cohen’s d value were 2.47 and 0.764, respectively, and most Cohen’s d values were higher than 0.8, revealing a large difference in the optical properties. When exposed to NaClO, some substances in the seed coat of maize may be oxidized and produce color differences compared with before oxidation. With the continuous production of new metabolites, the color difference caused by the oxidation of the maize surface was gradually diluted. Therefore, sample 1 (untreated) was used as a representative of sample 1 to explore the optical properties of *F. graminearum*-contaminated maize.

### 3.4. Exploration of Optical Properties

#### 3.4.1. Comparison and Selection of Different Optical Systems

**Figure 6:**
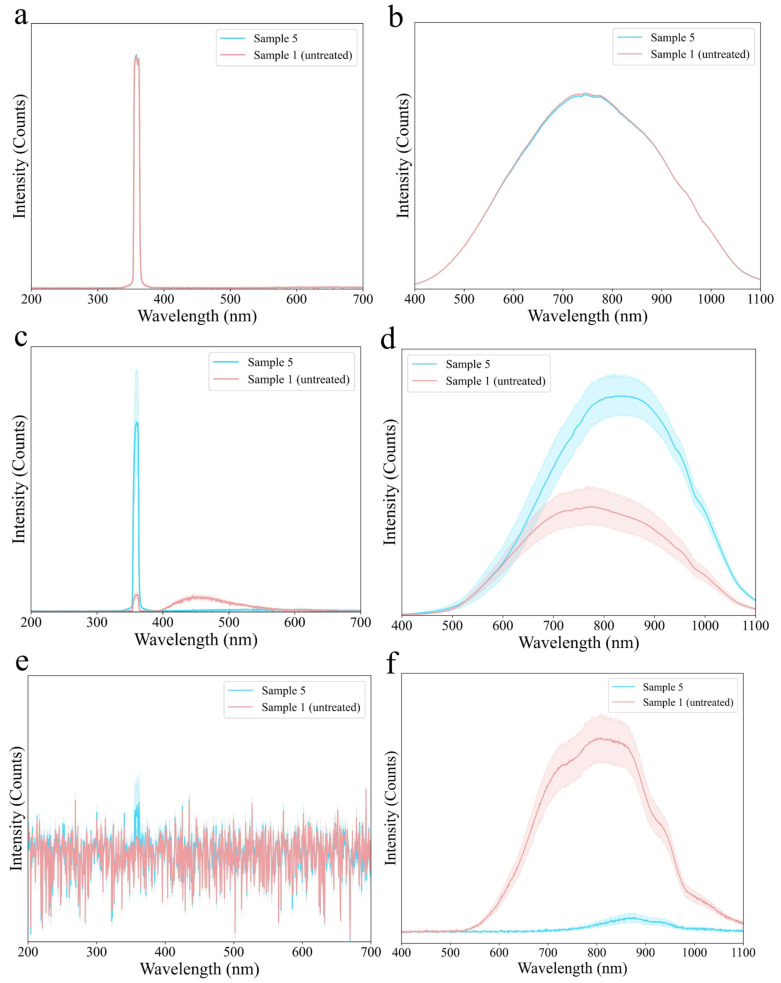
Average reflection spectra of RSS based on UV laser and integrating sphere (**a**), RSS based on VIS/NIR light source and integrating sphere (**b**), RSS based on UV laser and bifurcated optical fiber (**c**), and RSS based on VIS/NIR light source and bifurcated optical fiber (**d**). Average transmission spectra of TSS based on UV laser (**e**) and VIS/NIR light source (**f**).

In order to accurately reflect the optical properties of maize contaminated by *F. graminearum*, sample 1 (30 kernels) and sample 5 (30 kernels) were used for preliminary screening of optical systems with significant differences from four RSS and two TSS. Six reflection and transmission spectra were shown in Figure 6. For RSS based on the integrating sphere (Figure 6a,b), the spectral curve of sample 1 differed little from that of sample 5 in either UV or VIS/NIR bands. Due to the small volume of maize, the optical signal received by the spectrometer from maize was extremely weak, which made it difficult for integrating sphere-based RSS to be used to explore the optical properties of *F. graminearum*-contaminated maize. When using RSS based on bifurcated optical fiber (Figure 6c,d), the spectral curves of the two samples showed significant differences. The absorption capacity of sample 1 for UV and VIS/NIR bands was stronger than that of sample 5. It is worth noting that normal maize absorbing UV light would produce the excitation peak in the VIS band, while severely contaminated maize hardly produced excitation peaks in the VIS band, which revealed that RSS based on bifurcated optical fiber can be used to understand the optical properties of *F. graminearum*-contaminated maize. For TSS, the results showed that UV light could pass through sample 5, while VIS/NIR light could easily pass through sample 1. At a high excitation power density, sample 5 only generated a small peak area at 360 nm, indicating a small difference between normal maize and severely contaminated maize. For mildly contaminated samples, the excitation peak area was smaller and thus easily overlapped with spectral noise. Meanwhile, due to the high energy consumption and risk at high excitation power, UV-based TSS was not selected for further exploration of transmission optical properties. For TSS based on a VIS/NIR light source, the peak area of sample 1 was much larger than that of sample 5, even at a lower excitation power density. Therefore, TSS based on VIS/NIR light sources can be further used to explore the transmission optical properties of maize.

RSS was mainly used to explore the optical properties of maize surfaces, which was made possible by the accumulation of some mycotoxins in and under the maize seed coat. TSS could reflect the optical properties of the whole maize, including the embryo, endosperm, and seed coat, among which the embryo easily becomes a breeding ground for fungal spores due to its rich nutrition. Furthermore, the combination of RSS and TSS can better analyze the difference in optical properties between normal maize and *F. graminearum*-contaminated maize.

#### 3.4.2. Optical Properties of *F. graminearum*-Contaminated Maize

**Figure 7:**
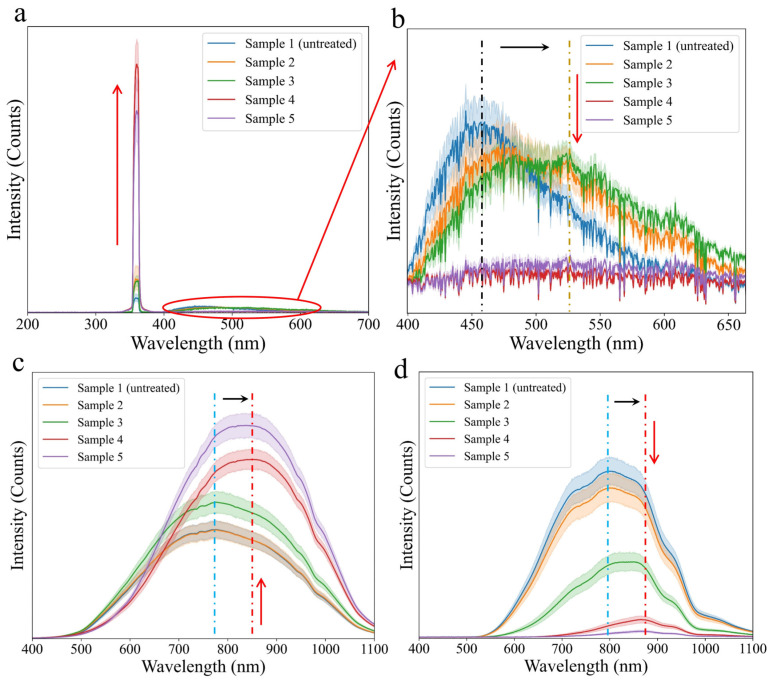
(**a**) Average UV excitation reflection spectra of five samples. (**b**) Magnified average UV excitation reflection spectra of five samples at the wavelength of 400–650 nm. (**c**) Average VIS/NIR reflection spectra of five samples. (**d**) Average VIS/NIR transmission spectra of five samples.

Figure 7a,b show the average UV excitation reflection spectrum for each sample. The spectral curves of the five samples between 300 and 400 nm had similar shapes. However, there were differences in intensity and peak location between these samples in bands between 400 and 600 nm. In detail, with the increasing severity of fungal contamination in maize, the intensity of the UV signal at 360 nm gradually increased, while the intensity at 400–600 nm showed a decrease and peak redshift phenomenon. Compared with the normal sample, the position of the excitation peak of mildly contaminated samples gradually shifted from 455 to 525 nm. However, for severely contaminated samples, the excitation peak gradually disappeared. According to the literature, *F. graminearum* metabolizes mycotoxins by consuming nutrients in maize during its growth, and ZEN is a fluorescent toxin [5,30]. For *F. graminearum*-contaminated maize, the decrease in peak redshift and peak intensity was mainly related to the accumulation of metabolic toxins (ZEN, etc.), fungal hyphal attachment, and nutrient loss. With the white hyphae covering the surface of the maize, the hyphae had a certain obstruction effect on the UV light, which would make the UV light more easily reflected to the receiving hole of the probe. The increasing intensity of maize samples at 360 nm further proved the reflection effect of hyphae on UV light. Due to the less UV light entering the seed coat, the excitation peak between 450 and 600 nm of severely contaminated maize was not obvious. Therefore, the rational use of UV excitation reflection optical properties is helpful to the development of online and low-cost detection equipment for *F. graminearum*-contaminated maize.

Figure 7c shows the average VIS/NIR reflectance spectrum for each sample. The normal and mildly contaminated samples had similar shapes on the spectral curves, where the curves of samples 1 and 2 almost coincide. The signal intensity of sample 3 increased significantly in the range of 500–1100 nm, but the peak did not shift. Compared with the normal sample, the severely contaminated samples not only had a significant increase in the intensity of the peak, but also the position of the peak gradually moved from 780 to 850 nm. The gradual increase in the reflection intensity of the spectral curve represented a decrease in VIS/NIR light signals to enter the seed coat, while the peak redshift represented an increase in metabolites containing auxochrome. Although the light-blocking performance of fungal hyphae was weaker than that of UV light, the spectral curves of normal samples and severely contaminated samples still showed significant differences with the increase of fungal hyphae. Auxochrome mainly refers to the functional groups that change the absorption position and increase the absorption intensity of the original molecules [53], such as -OH and -NH_2_. *F. graminearum* will metabolize in maize and produce a large number of toxins, among which DON and ZEN contain some auxochrome. When the electron-donating group is connected to the conjugated system in the molecule, the mobility of the electron cloud of the conjugated system increases. Subsequently, the energy level difference of the π → π* transition in the molecule is reduced, which eventually leads to the red shift of the peak [54]. It is worth noting that sample 1 and sample 2 cannot be effectively distinguished by VIS/NIR (400–1100 nm) reflectance spectra, which may be due to trace metabolic toxins and few hyphae in the seed coat. The grain color sorter can be used to screen out maize with serious mildew only using VIS light, but the identification effect on maize in the early and middle stages of mildew is not good. The results of the reflectance spectrum can explain the working principle of the color sorter in identifying *F. graminearum*-contaminated maize. Furthermore, the optical properties of VIS/NIR reflection combined with non-destructive detection methods are helpful to further improve the detection accuracy of fungal-contaminated maize.

Figure 7d shows the average VIS/NIR transmission spectrum of each sample. The transmission spectral curves and reflection spectral curves had similar change rules, including intensity changes and peak redshift. The difference from the reflection spectrum was that the transmission spectrum between the samples had a more significant difference. The average intensity of sample 2 was slightly lower than that of sample 1, but the standard deviations of the two samples were similar. With the increase of fungal growth and ZEN concentration, the average intensity of sample 3 between 500 and 1100 nm decreased significantly, and the peak red shift occurred. For severely contaminated samples, the average intensity continued to decline, and the redshift phenomenon became prominent. Sample 5 could hardly penetrate VIS/NIR light (400–1100 nm) when mycelia overgrew and ZEN overaccumulated. Like the factors affecting the reflection optical properties, *F. graminearum* hyphae and auxochrome are also two main factors affecting the change of transmission optical properties. The self-factors (thickness, shape, etc.) of maize may also affect the transmission optical properties, especially maize whose mycotoxin concentration just exceeds the national limit standard. However, the effect of *F. graminearum* hyphae and its metabolic toxins on transmission optical properties was more significant than that of maize’s self-factors. Thus, the transmission optical properties can be considered as a viable reference principle for developing online, rapid, and non-destructive testing equipment.

#### 3.4.3. Development of a Detection Platform for Single-Kernel Maize Using Optical Properties of *F. graminearum*-Contaminated Maize

**Figure 8:**
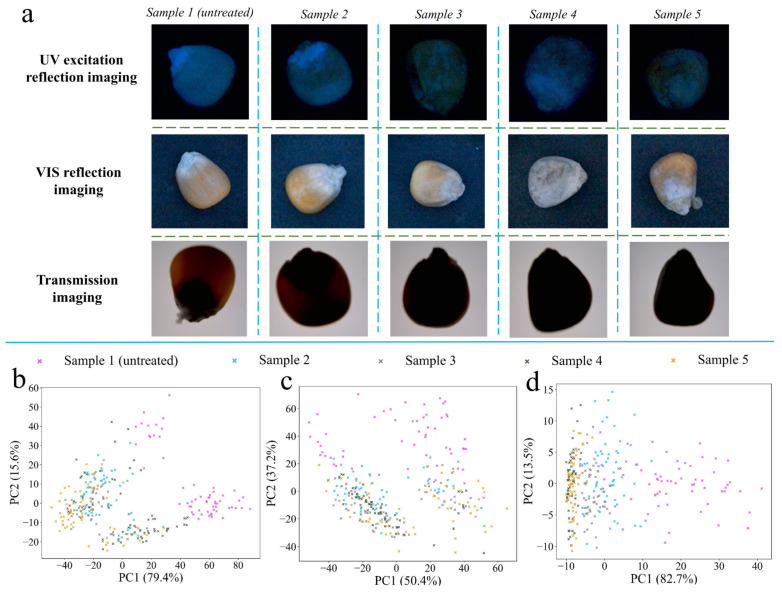
(**a**) Sample images collected under three imaging platforms. (**b**) PCA score plot of UV excitation reflection imaging. (**c**) PCA score plot of VIS reflection imaging. (**d**) PCA score plot of transmission imaging.

In order to understand the application feasibility of optical properties, this study attempted to combine the optical properties of maize with machine vision to develop three low-cost experimental platforms. The maize images collected under the three platforms were displayed in Figure 8a. From sample 1 to sample 5, the color intensity in the image showed a change in the rules, similar to that seen with the optical properties analyzed in Section 3.4.2. For UV excitation reflection imaging, the VIS intensity reflected by maize gradually decreased with the aggravation of fungal contamination. For VIS reflection imaging, the presence of white hyphae gradually increased the color intensity. In the case of transmission imaging, the transmitted light decreased significantly with the increase of fungal growth.

Figure 8b–d show the unsupervised classification effect of reducing the color features of different samples to the two-dimensional principal component space, corresponding to the UV excitation reflection image, VIS reflection image, and transmission image. When using the characteristics collected by the UV imaging platform, it could be observed that there was a certain separation trend between normal samples and mildly contaminated samples or severely contaminated samples. However, the characteristics of mildly contaminated and severely contaminated maize had a large number of overlaps in two-dimensional space. The VIS reflection imaging platform and transmission platform had the same change rule as the UV imaging platform in the principal component space. The main reasons for this are as follows: (1) The two dimensions of principal component 1 (PC1) and principal component 2 (PC2) contained fewer features, and the features extracted from maize were also less. (2) Considering the normal distribution of fungal contamination in maize, the small difference in color characteristics of adjacent samples resulted in a small difference mapped into two-dimensional space. (3) Because PCA is an unsupervised classification model, too many categories may not be conducive to the modeling process of PCA.

**Figure 9:**
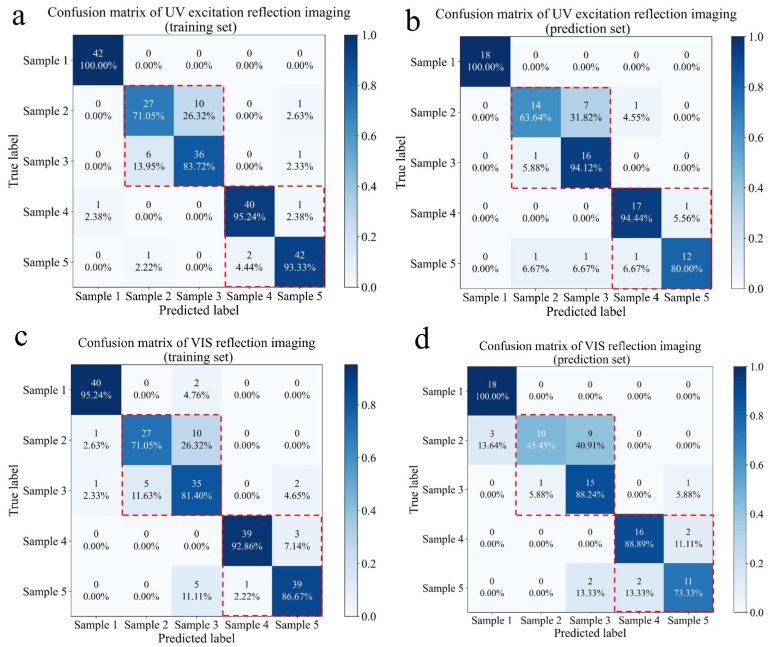

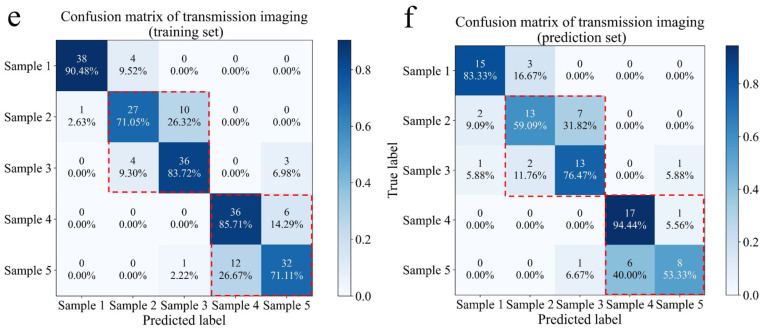
Confusion matrix based on UV excitation reflection imaging and SVM in the training set (**a**) and in the prediction set (**b**). Confusion matrix based on VIS reflection imaging and SVM in the training set (**c**) and in the prediction set (**d**). Confusion matrix based on transmission imaging and SVM in the training set (**e**) and in the prediction set (**f**).

Furthermore, SVM was used to analyze further the classification ability of the developed imaging platform for five samples (Figure 9). The similarity of the three imaging techniques was that the SVM model had the lowest classification accuracy for sample 2, whether in the training set or the prediction set. At the same time, there were many misclassifications in mildly contaminated samples (samples 2 and 3) or severely contaminated maize (samples 4 and 5). Therefore, it is not easy to achieve high-precision classification of five *F. graminearum* contamination levels using low-cost imaging platforms. If the classification level was reduced from five categories to three categories, the classification accuracy of SVM for normal, mildly contaminated, and severely contaminated samples would be significantly improved. SVM based on UV excitation imaging had the highest classification accuracy, and its classification accuracy for three levels of maize was more than 93%, both on the training set and the prediction set. Although the classification accuracy of VIS reflection imaging and transmission imaging was lower than that of UV excitation imaging, the classification accuracy of *F. graminearum*-contaminated maize could exceed 80%. Significantly, the cost of each imaging platform was less than USD 100, which reduced the cost of developing online multi-channel detection equipment for *F. graminearum*-contaminated maize. Overall, a full understanding of the optical properties of fungal-contaminated maize is beneficial for the development of low-cost and high-throughput equipment.

Detection cost, detection speed, and detection accuracy are important indicators of the need to balance each other in the development of fungal-contaminated grain sorting equipment. The existing optical detection methods were mainly used for sampling detection of maize or had high instrument cost [25,27], and the optical characteristics of single-kernel *F. graminearum*-contaminated maize have not been systematically revealed. Due to the morphological differences among maize kernels, it is difficult to balance the weight of detection cost, detection speed, and detection accuracy. At present, imaging technology is one of the best methods to achieve rapid and high-throughput grain detection [55,56]. Thus, this study was to combine the optical properties and machine vision to improve the detection accuracy of *F. graminearum*-contaminated maize in the case of ensuring the detection speed.

The developed low-cost imaging platforms based on proven optical properties could assess the severity of *F. graminearum* contamination in maize. However, the application of a single technology was still limited, such as the grain color sorter based on the VIS reflective optical properties in practical applications. The combination of multiple technologies can be used as a feasible solution to improve the detection accuracy of fungal-contaminated maize, but the combination of technologies may lead to increased costs and slower speed of online detection. Extracting more features is also conducive to improving the detection accuracy of fungal-contaminated maize without increasing the cost and without affecting the online detection speed. Meanwhile, selecting more complex classification models is conducive to further improving the classification accuracy of fungal-contaminated maize, such as deep convolutional neural networks. In addition, optical methods such as Raman spectroscopy and near/mid-infrared spectroscopy (1100–2500 nm) can also be used to explore the optical properties of *F. graminearum*-contaminated maize. In general, a large number of detection platforms need to be applied to the detection equipment, and the sensitive band of a low-cost industrial camera is usually 400–1100 nm. Considering the cost of equipment, this study focused on the optical properties of low-cost imaging methods, and the developed experimental platform proved the availability of the obtained optical properties. Since the industrial camera is more sensitive to the VIS light than NIR light, the NIR light source is not selected as the experimental light source. Subsequent research can use NIR light sources to develop the optical imaging platform. In addition, the optical properties of maize were mainly affected by fungal hyphae and their metabolic toxins, and it had certain versatility for different maize varieties. However, there may be some differences between naturally contaminated maize and cultured-contaminated maize, including the growth position of fungal hyphae, the combined infection of multiple fungi, and the coexistence of multiple toxins. Therefore, it is necessary to study the optical properties of naturally contaminated maize.

#### 3.4.4. Feasibility Analysis of Asymmetric Polarization Technique to Enhance the Optical Properties of *F. graminearum*-Contaminated Maize

**Figure 10:**
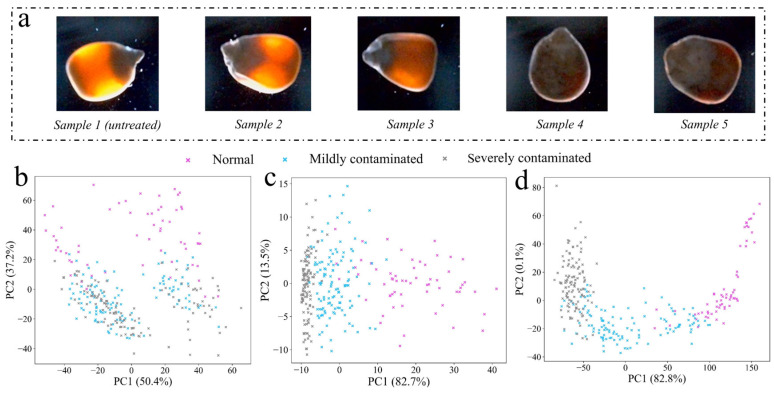
(**a**) Sample images collected using asymmetric polarization platform. (**b**) PCA score plot of VIS reflection imaging after reducing maize categories. (**c**) PCA score plot of transmission imaging after reducing maize categories. (**d**) PCA score plot of asymmetric polarization imaging after reducing maize categories.

Asymmetric polarization imaging technology was proposed as a tentative solution for reference to further improve the detection accuracy of optical imaging for *F. graminearum*-contaminated maize. Compared with transmission imaging, asymmetric polarization imaging had more distinct optical differences (Figure 10a), which was due to the effect of polarized light. At the same time, due to the asymmetric installation of the polarizer, it could be observed that the camera captured the light signal on the surface of the maize, especially the severely contaminated maize with low light transmittance. In the case of cereals, excessive classification is not of great significance for practical applications. As long as the vast majority of severely contaminated maize and some mildly contaminated maize can be identified and eliminated, the quality of maize will be greatly improved. Therefore, three contamination levels of maize were used to test the classification performance of asymmetric polarization imaging. After reducing the number of categories from five to three, it could be observed that the clustering effect of transmission imaging on the three levels of maize was significantly better than that of VIS reflection imaging using only two dimensions of feature vectors (Figure 10b,c). Compared with transmission imaging, the center distance of the three levels of maize samples was farther using asymmetric polarization imaging technology (Figure 10d), especially for normal samples and severely contaminated samples. The center distance had a positive correlation with the difference of color features between samples, indicating that asymmetric polarization imaging had better unsupervised classification ability than the other two imaging. The PC1 contribution rate of asymmetric polarized light imaging was 82.8%, while the contribution rate of PC2 was less than 1%, which may be due to the limited mildew information contained in the color features of asymmetric polarized images.

**Figure 11:**
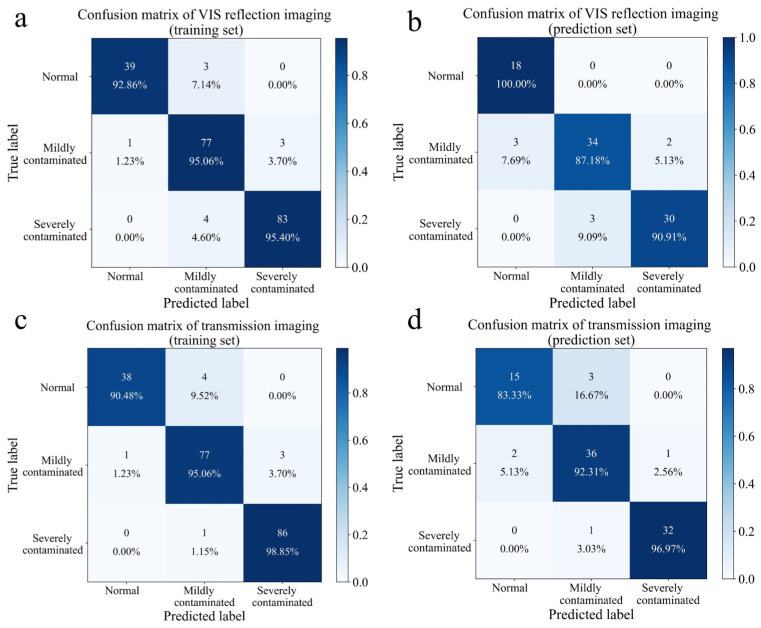

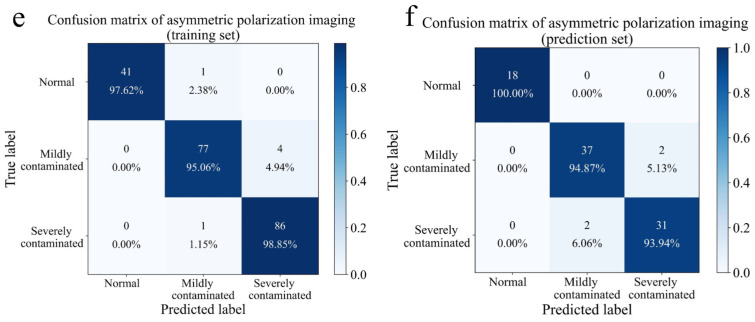
Confusion matrix (only three levels of maize) based on VIS reflection imaging and SVM in the training set (**a**) and in the prediction set (**b**). Confusion matrix (only three levels of maize) based on transmission imaging and SVM in the training set (**c**) and in the prediction set (**d**). Confusion matrix (only three levels of maize) based on asymmetric polarization imaging and SVM in the training set (**e**) and in the prediction set (**f**).

SVM was used to evaluate the classification performance of asymmetric polarization imaging technology for three levels of maize (Figure 11). In the training set, the classification accuracy of SVM-based reflection imaging and transmission imaging for the three levels of maize exceeded 90%, while the classification accuracy of SVM-based asymmetric polarization imaging for the three levels of maize exceeded 95%. When predicting the fungal contamination level of maize, only the classification accuracy of asymmetric polarization imaging for maize exceeded 93%, which was significantly better than the other two imaging methods. With the aggravation of fungal contamination, the hyphae on the surface of maize were obvious. Thus, future research can consider adding texture features to expand the dimension of data, which may help to increase the contribution rate of PC and further improve the classification accuracy of SVM. Polarized light imaging has been shown to be used for the detection of fungal contamination of maize [32,33], but complete polarization could not contain the reflected signal on the surface of maize. The asymmetric installation of polarizers was conducive to the retention of some reflected signals and had richer optical information. Overall, the introduction of asymmetric polarization technology is helpful in improving the classification accuracy of *F. graminearum*-contaminated maize, and it is expected to achieve the development of higher precision color sorting equipment.

## 4. Conclusions

This study investigated the reflection optical properties, transmission optical properties, and volatomics of *F. graminearum*-contaminated maize. The identified optical properties were helpful for the development of online and non-destructive equipment, and the known volatileomics could promote the application of olfactory sensors in maize quality detection. In the application of optical characteristics, the developed optical imaging platform showed excellent classification ability for *F. graminearum*-contaminated maize, especially when the classification category was less than or equal to three. However, maize is not only contaminated by *F. graminearum*, but also may be infected by complex fungi. In the future, it is necessary to study all the dominant fungal strains in maize and also to study the optical properties of maize under the infection of complex fungi, so as to accelerate the development of high-precision and low-cost equipment for fungal-contaminated maize. Overall, a full understanding of the optical properties and volatileomics of *F. graminearum*-contaminated maize can lay the foundation for the further development of non-destructive technology and provide a reference for the study of other fungal-contaminated maize.

## Figures and Tables

**Table 1:** Relatively high levels of 10 VOCs from normal and severely contaminated maize.

Volatile (Sample 1)	CAS	RI	Peak Area (% TIC)	Volatile (Sample 5)	CAS	RI	Peak Area (% TIC)
1-Pentanol, 3-methyl- (82%)	589-35-5	878	36.31	Acetic acid (86%)	64-19-7	[-]	69.87
Fenchone (82%)	126-21-6	1093	7.32	1-Butanol, 3-methyl- (81%)	123-51-3	[-]	7.91
1,2-Propanedione, 1-phenyl- (79%)	579-07-7	1148	6.87	Benzene, 1-ethyl-4-methoxy- (94%)	1515-95-3	1134	5.76
Nonanal (87%)	124-19-6	1116	5.03	Benzene, 4-ethyl-1,2-dimethoxy- (90%)	5888-51-7	1405 (-)	4.75
Cyclohexanol (80%)	108-93-0	886	1.72	Styrene (80%)	100-42-5	901	3.72
Linalool (80%)	78-70-6	1121	1.48	Heptanal (79%)	111-71-7	[-]	3.51
Carotol (89%)	465-28-1	1612	1.11	1-Butanol, 3-methyl-, acetate (80%)	123-92-2	892	1.44
1-Hexanol, 5-methyl-2-(1-methylethyl)- (81%)	2051-33-4	1201 (-)	1.09	Pentanoic acid, 3-methyl- 78%	105-43-1	928	0.48
1-Octyn-3-ol (78%)	818-72-4	1085 (-)	0.80	3-Octanone 78%	106-68-3	987	0.40
1-Octen-3-ol (79%)	3391-86-4	959	0.73	Furan, 2-pentyl- 79%	3777-69-3	994	0.21

TIC—total ion chromatogram; RI—retention indices; CAS—Chemical Abstract Service registry number. [-] represents incalculability and (-) represents that RI does not exist in the NIST database.

**Table 2:** Mann–Whitney U test for sample 1 (untreated), sample 1 (sterilized with NaClO), and sample 1 (cultured for five days).

Independent Samples		Median		Statistics	*p*	Median Difference	Cohen’s d Value
Group A	Group B	Group A	Group B				
Untreated_B	Sterilized_B	121.936	132.535	259	0.009 ***	10.599	0.837
Untreated_B	FiveDays_B	121.936	122.709	412	1.148	0.773	0.204
Sterilized_B	FiveDays_B	132.535	122.709	634	0.013 **	9.826	0.764
Untreated_G	Sterilized_G	128.998	142.112	133	0.000 ***	13.113	1.452
Untreated_G	FiveDays_G	128.998	126.704	484	1.230	2.294	0.112
Sterilized_G	FiveDays_G	142.112	126.704	791	0.000 ***	15.408	1.604
Untreated_R	Sterilized_R	129.812	152.842	38	0.000 ***	23.03	2.47
Untreated_R	FiveDays_R	129.812	130.785	384	0.658	0.974	0.261
Sterilized_R	FiveDays_R	152.842	130.785	842	0.000 ***	22.057	2.241

**—5% significance level, ***—1% significance level. Untreated_x-x channel of image for sample 1 (untreated). Sterilized_x-x channel of image for sample 1 (sterilized with NaClO). FiveDays_x-x channel of image for sample 1 (cultured for five days).

## Data Availability

The original contributions presented in the study are included in the article/Supplementary Materials, further inquiries can be directed to the corresponding authors.

## Supplementary Materials

The following supporting information can be downloaded at: https://www.mdpi.com/article/10.3390/foods13193087/s1, Figure S1: Molecular structures of 11 VOCs from normal (sample 1) and severely contaminated maize (sample 5); Table S1: Relatively low or low confidence levels of VOCs from normal and severely contaminated maize.
