# Supplementary material for: Exploration of Volatileomics and Optical Properties of Fusarium graminearum-Contaminated Maize: An Application Basis for Low-Cost and Non-Destructive Detection
Source: Foods. 2024 Sep 27;13(19):3087. doi: 10.3390/foods13193087 (PMC11475652; doi:10.3390/foods13193087)
Supplement: Supplementary file 1 [file foods-13-03087-s001.zip › foods-3224653-supplementary.pdf]

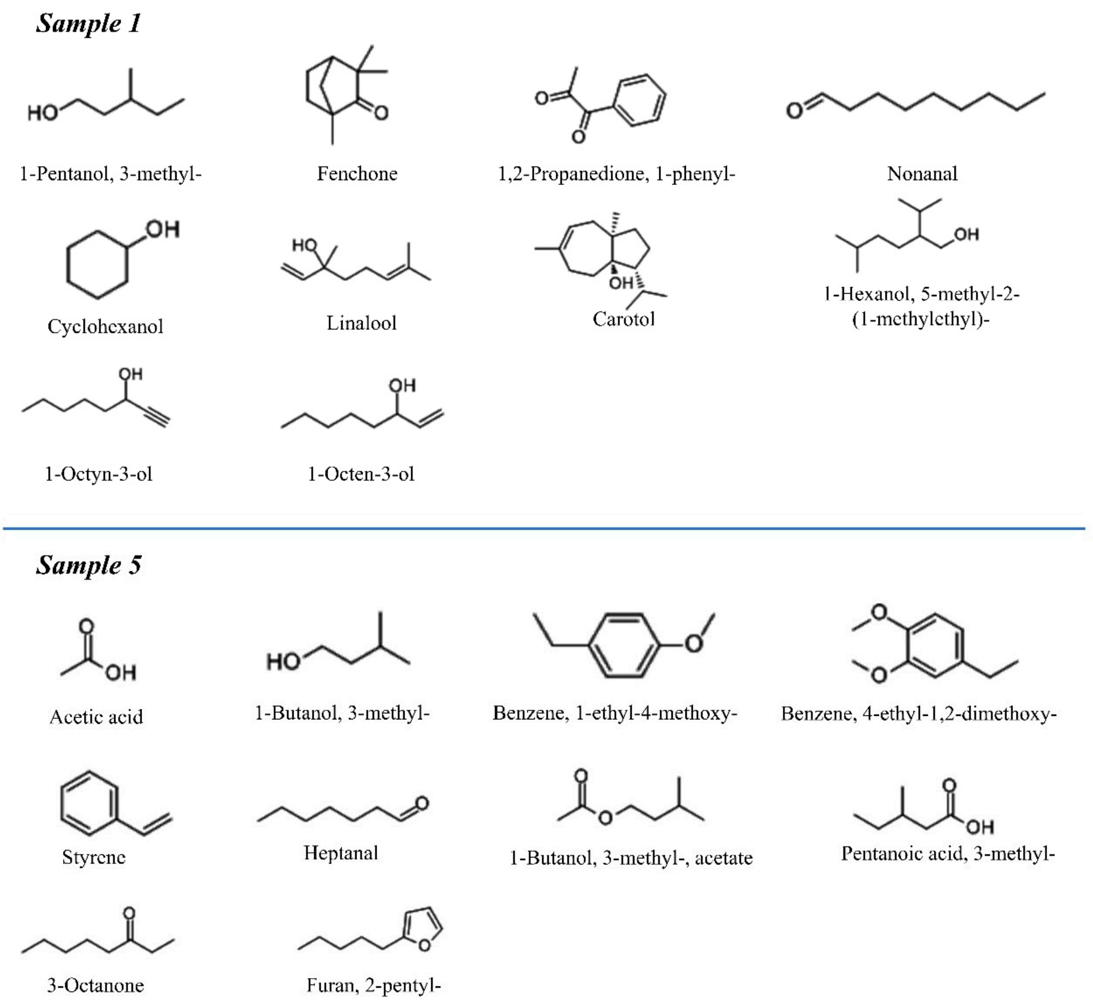

Figure S1. Molecular structures of 11 VOCs from normal (sample 1) and severely contaminated maize (sample 5).

Table S1. Relatively low or low confidence levels of VOCs from normal and severely contaminated maize

Votatile (sample 1)	CAS	Peak area (% TIC)	Votatile (sample 5)	CAS	Peak area (% TIC)
2-Decen-1-ol, (E)-	18409-18-2	11.90	6-Methyl-3,5-heptadiene-2-one	1604-28-0	0.63
1,3-Butanediol, (S)-,	24621-61-2	6.95	p-Xylene	106-42-3	0.38
2,2-Dimethyl-1,3-butanediol	76-35-7	2.00	1,2,4-Benzenetricarboxylic acid, 1,2-dimethyl ester	[-]	0.20
1-Octanol, 2,7-dimethyl--	15250-22-3	1.70	Butanoic acid, 2-methyl-	116-53-0	0.19
1-Octyn-3-ol, 4-ethyl-	5877-42-9	1.41	2-Hexanol, 5-methyl-	627-59-8	0.18
4,4-Dimethyl-cyclohex-2-en-1-ol	[-]	1.15	3-Octanol, acetate	4864-61-3	0.18
2-Propanone, 1-methoxy- 78%	5878-19-3	1.14	2-Heptanol, acetate	5921-82-4	0.08
Carbonic acid, nonyl prop-1-en-2-yl ester	[-]	0.93	Undecanal	112-44-7	0.07

2-Furanmethanol, 5-ethenyltetrahydro-.alpha.,.alpha.,5-trimethyl-, cis-	5989-33-3	0.84	Benzene, 4-ethenyl-1,2-dimethoxy-	6380-23-0	0.04
Butanoic acid, 4-hydroxy-	591-81-1	0.82			
1-Octanol, 2-butyl-	3913-02-8	0.72			
1-Decanol, 2-ethyl-	21078-65-9	0.69			
1-Decanol, 2-hexyl-	2425-77-6	0.64			
trans-Linalool oxide (furanoid)	34995-77-2	0.60			
Carbonic acid, decyl undecyl ester	[-]	0.59			
2,4-Di-tert-butylphenol	96-76-4	0.58			
1-Octanol	111-87-5	0.56			
2-Heptenoic acid, octyl ester	[-]	0.54			
Cyclopropanemethanol, 2-methyl-2-(4-methyl-3-pentenyl)-	[-]	0.50			
Phenol, 3,5-bis(1,1-dimethylethyl)-	1138-52-9	0.48			
(R)-(-)-(Z)-14-Methyl-8-hexadecen-1-ol	30689-78-2	0.41			
Cyclohexanol, 2-methyl-5-(1-methylethenyl)-, (1.alpha.,2.alpha.,5.beta.)-	18675-33-7	0.40			
Acetic acid, pentyl ester	628-63-7	0.28			
Fenchyl acetate	13851-11-1	0.25			
Dodecanal	112-54-9	0.20			
2-Nonenal, (E)-	18829-56-6	0.19			
2-Isopropyl-5-methyl-1-heptanol	[-]	0.18			
Decanal	112-31-2	0.17			
Heptanal	111-71-7	0.14			
Azulene	275-51-4	0.12			
Chloroacetic acid, nonyl ester	5451-96-7	0.08			
Oxalic acid, allyl pentadecyl ester,	[-]	0.07			
4-Ethyl-1-hexyn-3-ol	[-]	0.06			
Anethole	4180-23-8	0.06			
Oxalic acid, 6-ethyloct-3-yl heptyl ester	[-]	0.06			
Oxalic acid, allyl hexadecyl ester	[-]	0.05			
Oxalic acid, allyl nonyl ester	[-]	0.04			
2,4-Pentadien-1-ol, 3-pentyl-, (2Z)-	[-]	0.03			
